# Adaptational changes in physiological and transcriptional responses of *Bifidobacterium longum* involved in acid stress resistance after successive batch cultures

**DOI:** 10.1186/s12934-019-1206-x

**Published:** 2019-09-12

**Authors:** Yanxia Wei, Jing Gao, Dianbin Liu, Yang Li, Wenli Liu

**Affiliations:** Jiangsu Key Laboratory of Immunity and Metabolism, Laboratory of Infection and Immunity, Department of Pathogenic Biology and Immunology/School of Stomatology, Xuzhou Medical University, Xuzhou, 22104 Jiangsu, China

**Keywords:** Bifidobacteria, Adaptive evolution, Acid stress, Cross-protection, Fatty acid

## Abstract

*Bifidobacterium* inhabiting the human and animal intestinal tract is known for its health-promoting effect. Tolerance to acid stress is crucial for bifidobacteria to survive and then exert their beneficial effects in the gut. A long-term adaptation in successive batch cultures was used as evolutionary engineering strategy to improve acid stress tolerance in an industrial probiotic strain, *B. longum* JDM301. Its derivative, JDM301AR showed higher resistance to several stress conditions, including acid stress than the parental strain, JDM301. To better understand bifidobacterial acid stress response, the changes of fatty acid (FA) in cell membrane of these two strains were determined. A shift in the production of FA in cell membrane, characterized by increased C14:0 was found, when JDM301AR was exposed to low-pH environment. It was implied that the increased production of C14:0 is associated with the acquisition of acid-tolerant phenotype for JDM301AR. High-throughput RNA-sequencing was performed to analyze the changes of gene expression profile after acid-exposure. The transcriptional profiles of JDM301AR and JDM301 under normal condition and acid stress were compared to reveal the different acid response between them. A total of 5 genes involved in FA metabolism were upregulated and no downregulated genes were found in response to acid stress in JDM301AR. The up-regulated BLJ_0565 and BLJ_1105 may play important roles in the modification of membrane FA composition of JDM301AR after acid exposure. Overall, these results suggested that successive batch cultures induced the acid stress tolerance of *B. longum* involved in transcriptional and physiological responses, including modification of cell wall and cell membrane, metabolism of amino acid and neutralization of internal pH by strengthening NH_3_ production and transport.

## Background

Bifidobacteria are anaerobic and Gram-positive bacteria mainly inhabiting the human and animal intestinal tract. It was believed that bifidobacteria can strengthen the intestinal barrier, modulate the immune response, inhibit pathogens and so on [1]. However, during the industrial manufacturing processes and passage through the digestive tract of the host, bifidobacteria encounter various types of stress, which can reduce bifidobacterial viability and probiotic effects. In particular, bifidobacteria used as probiotics, need to survive the acid barrier of the stomach [2]. The acid barrier represents a major challenge for probiotics. The results of clinical trials implied a direct dose–effect correlation with probiotic efficacy [3]. Overall, acid tolerance is critical to bifidobacterial survival and therefore to the functionality of various probiotic products.

The resistance of bacteria to lethal stress can be improved by application of sub-lethal stress during culture, which called stress adaptation. The acid adaptation has been reported in *Bifidobacterium longum* (*B. longum*) and *Bifidobacterium animalis* (*B. animalis*) and in several other gram-positive bacteria such as *Streptococcus mutans*, *Enterococcus faecalis* [4–7]. In previous reports, *B. longum* 8809dpH and BBMN68 mutant strain showing enhanced acid tolerance compared with the wild-type strain were isolated by culturing cells in low pH pressure achieved through modified medium with hydrochloric acid (HCl) [5]. Although acid tolerance response of *B. longum* has been studied in previous reports, the adaptation mechanisms revealing acid stress response in bifidobacteria are still largely unknown.

In our study, the acid tolerance of *B. longum* JDM301, an industrial probiotic strain was improved only by successive batch cultures in normal condition [cultured anaerobically in De Man-Rogosa-Sharpe (MRS) supplemented with 0.05% (w/v) l-cysteine-HCl at an initial pH of 6.5 at 37 °C]. This study provided an approach to improve bifidobacterial tolerance response in normal condition and a first insight into the stress cross-protection, the alterations of the gene expression patterns and the changes in cell membrane lipids of industrial bifidobacterial strain achieved by adaptive evolution through successive batch cultures without additional stress. The purpose of this study was to investigate the transcriptional and physiological responses of two closely related strains of *B. longum* JDM301 (JDM301), a widely used strain in China [8] and its acid-resistant derivative *B. longum* JDM301AR (JDM301AR), a strain obtained by successive batch cultures of JDM301. The different responses of the acid-resistant strain was compared with its parental strain, JDM301. To reveal the differences of gene transcription between JDM301 and JDM301AR potentially responsible for acid resistance and the cross-protection, the transcriptional response profiles of JDM301 and JDM301AR cultured under normal condition were analyzed and compared. To further reveal the mechanism underling the acid resistance in JDM301AR, JDM301 and JDM301AR were exposed to acidified culture medium and the gene transcription of each strain in acid condition was compared with that in normal condition, respectively. *B. longum* JDM301 was chosen based on its wide use in industry as probiotic in China and the availability of complete genome sequence information [9] to reveal the adaptive evolution of bifidobacteria through successive batch cultures in normal condition.

## Results and discussion

### Enhancement of the stress tolerance of JDM301 after adaptive evolution

**Fig. 1:**
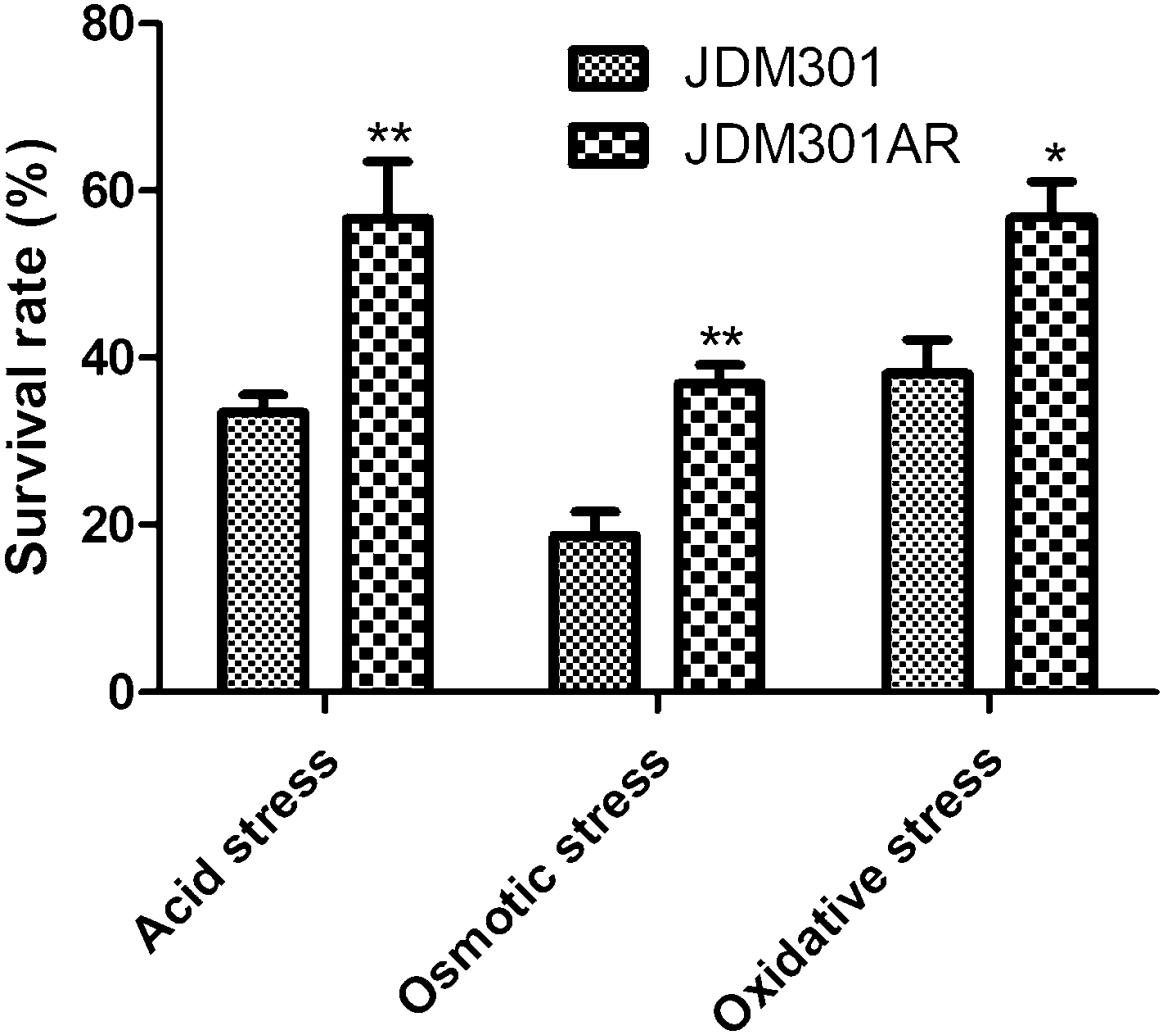
Survival of *Bifidobacterium longum* JDM301 and JDM301AR under acid stress, osmotic stress or oxidative stress. Exponential cells were collected and resuspended into fresh modified MRS with low pH (3.5), or into modified MRS containing 1.25 mM H_2_O_2_ (oxidative stress) or 0.5 M NaCl (osmotic stress). Significant differences between JDM301 and JDM301AR under different conditions were marked. *P < 0.05; **P < 0.01

To study the effects of successive batch cultures (150th repeats) on the stress tolerance of *B. longum*, cells were collected and transferred into acidified MRS medium (pH = 3.5), MRS with 0.5 M sodium chloride (NaCl) or containing 1.25 mM hydrogen peroxide (H_2_O_2_). The cells (JDM301AR) showed significantly higher survival rate than the non-adapted strain (JDM301) after 1.5 h incubation under acidified medium. Furthermore, JDM301AR displayed higher tolerance to osmotic and oxidative stress, when compared with JDM301 (Fig. 1). Cross-protection response has been reported in several other bacteria, such as *Escherichia coli*, *Vibrio harveyi* and *Lactococcus lactis* [10–12]. A cross-protection mechanism between bile and acid stress was discovered in *B. animalis* [13]. The results in our study showed that successive batch cultures provided this bacterium with cross-protection against several stress conditions [14].

### Differences of gene transcription profile between JDM301 and JDM301AR under normal condition

**Fig. 2:**
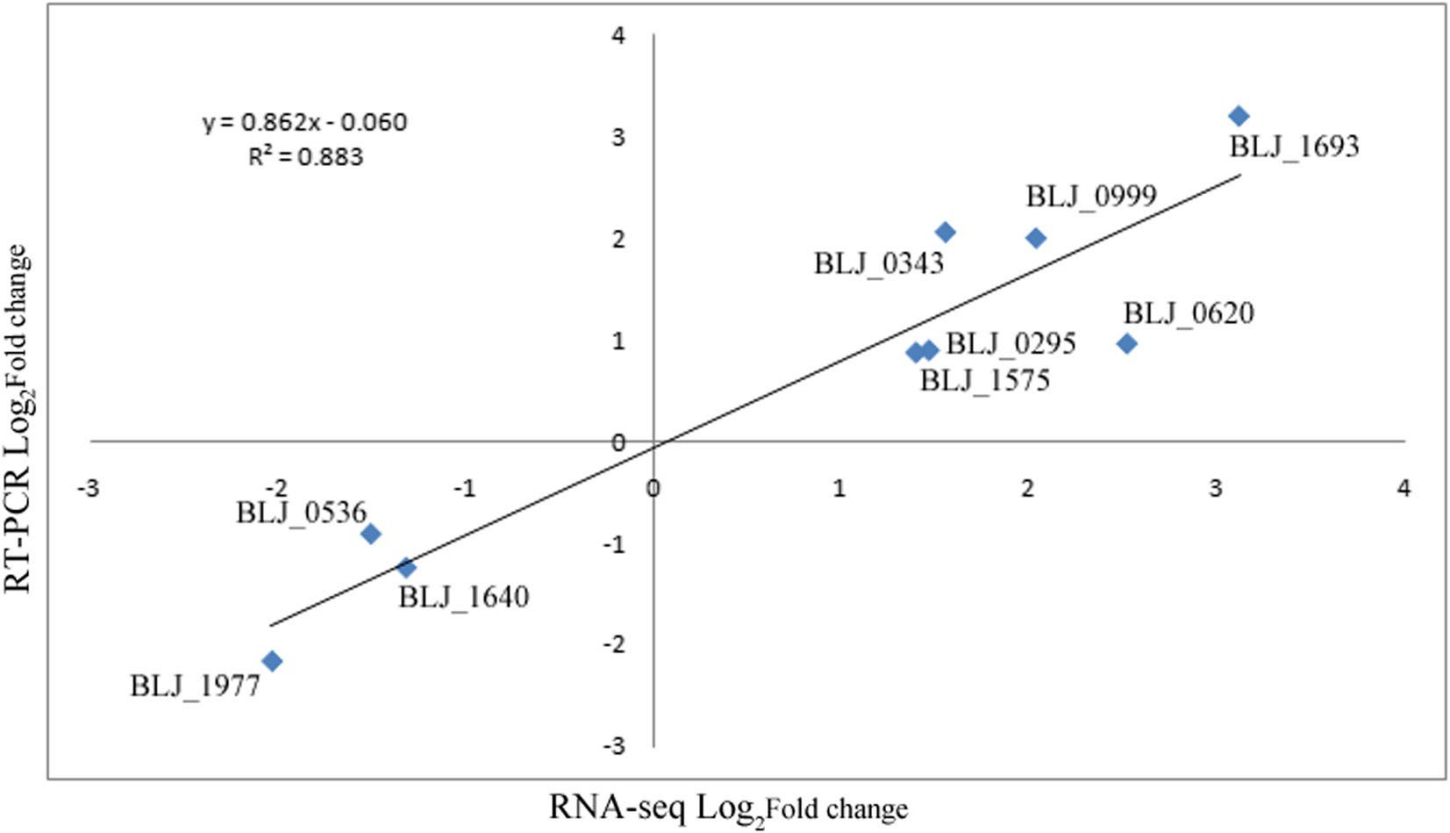
Correlation of fold change values from RNA-Seq and real-time quantitative PCR results (RT-PCR). The best-fit curve was shown along with the calculated equation and r^2^ value

**Fig. 3:**
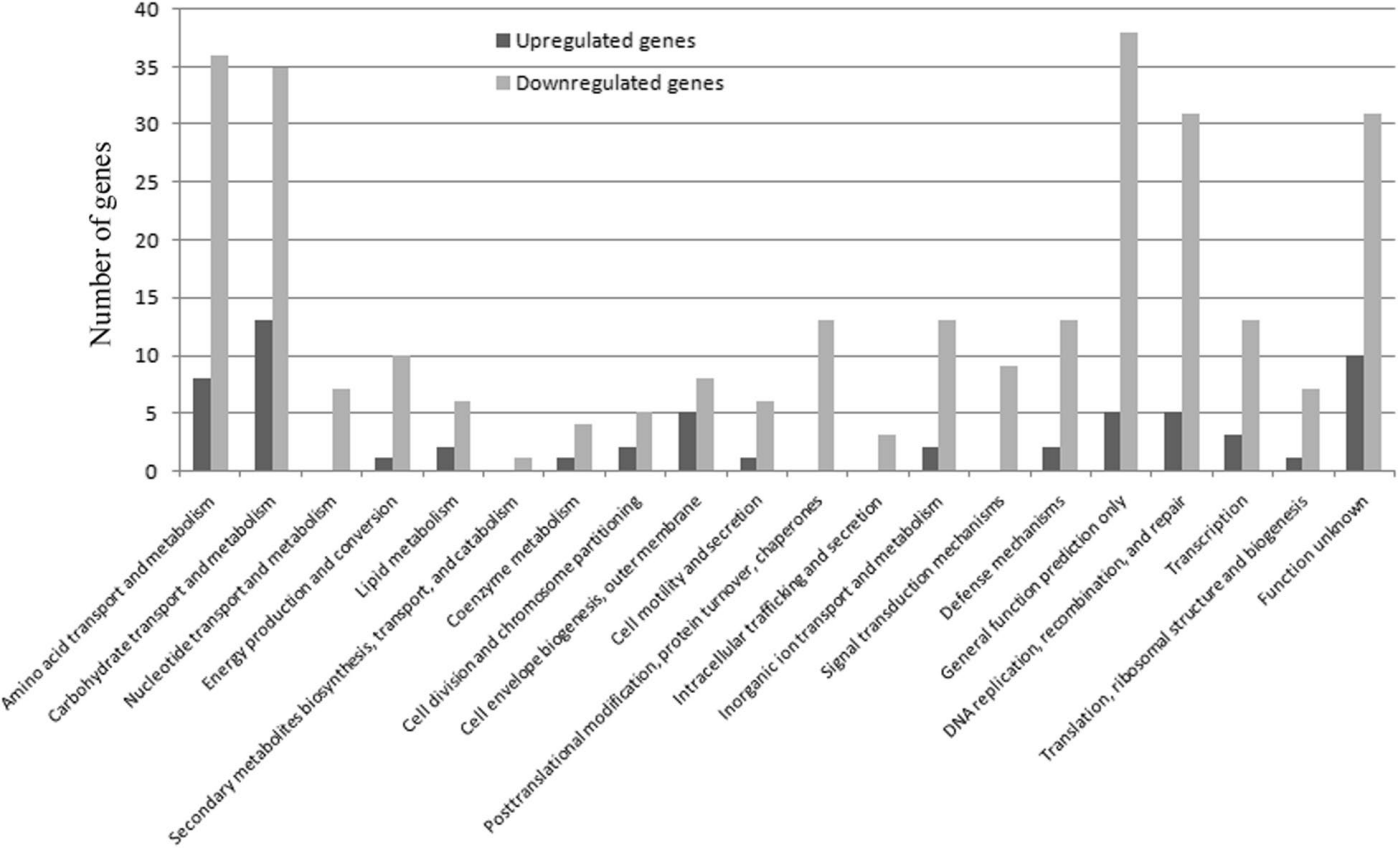
Numbers of genes showing different expression in JDM301AR compared with JDM301 in normal condition. These genes were significantly upregulated (black bars) or downregulated (gray bars) in JDM301AR compared with JDM301 in normal condition and grouped according to functional category

Acid tolerance is critical to bifidobacterial survival and their functionality. Thus, to explore the genes involved in the enhanced acid tolerance response and the cross-protection mechanism of JDM301AR, the transcriptional response profiles of JDM301 and JDM301AR were analyzed and compared under normal condition (cells were inoculated in modified MRS at an initial pH of 6.5 without stress and cultured under anaerobic condition at 37 °C). A strong positive correlation (R^2^ = 0.883) between RNA sequencing (RNA-Seq) data and real time quantitative polymerase chain reaction (RT-PCR) data was showed in Fig. 2. JDM301AR showed a total of 350 differentially expressed (DE) genes after adaptive evolution through successive batch cultures. Among the DE genes, 289 (82.6%) were downregulated, while 61 (17.4%) were upregulated (Fig. 3). Among them, BLJ_1400 (Log_2_FC = 1.6) encoding cystathionine gamma-synthase (MetC_3_) was upregulated in JDM301AR (Additional file 1). MetC_3_ can catalyze the combination of cysteine with succinylhomoserine to produce cystathionine [15]. Differently from the gene involved in production of cystathionine, genes encoding cystathionine lyase (BLJ_1778, Log_2_FC = − 10.1 and BLJ_1779, Log_2_FC = − 9.0) were all significantly downregulated (Additional file 1). The cystathionine can be converted to ammonia (NH_3_), l-cysteine and α-ketobutyrate by cystathionine gamma lyase (BLJ_1779) or NH_3_, l-homocysteine and pyruvate by cystathionine beta-lyase (BLJ_1778), when needed. Additionally, the cystathionine beta-lyase can catalyze the production of NH_3_ from l-cysteine [16]. NH_3_ can enhance the acid tolerance of bacteria by neutralizing H^+^. These results indicated that the ability to produce NH_3_ is promoted in JDM301AR by strengthening the NH_3_ reservation through accumulation of cystathionine. Borja et al. showed that the production of cystathionine gamma-synthase increased in an acid-resistant mutant obtained by incubated bifidobacteria at low pH medium [5], while the downregulation of cystathionine lyase has not been reported in acid tolerance response of bifidobacteria. Taken together, these results suggested that the ability to produce NH_3_ is promoted in JDM301AR by strengthening the NH_3_ reservation through accumulation of cystathionine, which was achieved through up-expression of MetC_3_ and down-expression of cystathionine lyase.

A gene cluster for pantothenate and CoA (Coenzyme A) biosynthesis (BLJ_0525-531) was upregulated in JDM301AR compared with JDM301 (Additional file 1). CoA is essential for central metabolism, including the degradation and synthesis of fatty acid (FA) and phospholipids synthesis [17]. CoA is derived from pantothenate and essential for the growth of organisms [18]. Reports showed that CoA biosynthesis played important roles in regulation of oxidative and osmotic stress response and that CoA also affected acid stress response by regulating protein acetylation [19–22]. These results suggested that the gene cluster may play important roles in the cross-protection of JDM301AR.

Furthermore, another gene cluster (BLJ_1300-1303) involved in peptidoglycan synthesis was also upregulated in JDM301AR (Additional file 1). Peptidoglycan is responsible for the rigidity of the bacterial cell wall, which is needed to cope with both high turgor pressure and environmental stress [23, 24]. It could be referred that strengthening the cell wall by enhancing peptidoglycan synthesis is one of the strategies of JDM301 and JDM301AR to response to adverse stress, including acid stress. These results implied that the enhanced synthesis of CoA and peptidoglycan play important roles in the cross-protection and the enhanced resistance of JDM301AR to acid stress.

The expression of 7 genes involved in cell division and chromosome partitioning was changed (Additional file 1). Among them, FtsZ (BLJ_0917, Log_2_FC **= − **2.4) and other 4 proteins were downregulated. Only two proteins, SOS-response cell division inhibitor, SulA encoded by BLJ_1879 (Log_2_FC = 1.9) and an ATPase involved in chromosome partitioning encoded by BLJ_0570 (Log_2_FC = 1.1) were upregulated. SulA can inhibit FtsZ polymerization [25]. In previous reports, some bacteria survived various environmental stress, such as antibiotic treatment and oxidative stress by slowing growth down or growth arrest [26, 27]. These results implied that the potentially decreased cell growth or division by down-regulating FtsZ may be helpful for *Bifidobacterium* to survive acid stress. Furthermore, SOS-response transcriptional repressors (LexA) encoded by BLJ_0655 (Log_2_FC = 1.1) was also upregulated. The two pieces of evidence indicated that the assembly of the Z ring may be prevented partially by upregulated SulA to protect daughter cells during cell division from the premature segregation of damaged DNA.

### Responses induced at low pH in JDM301 and JDM301AR

**Fig. 4:**
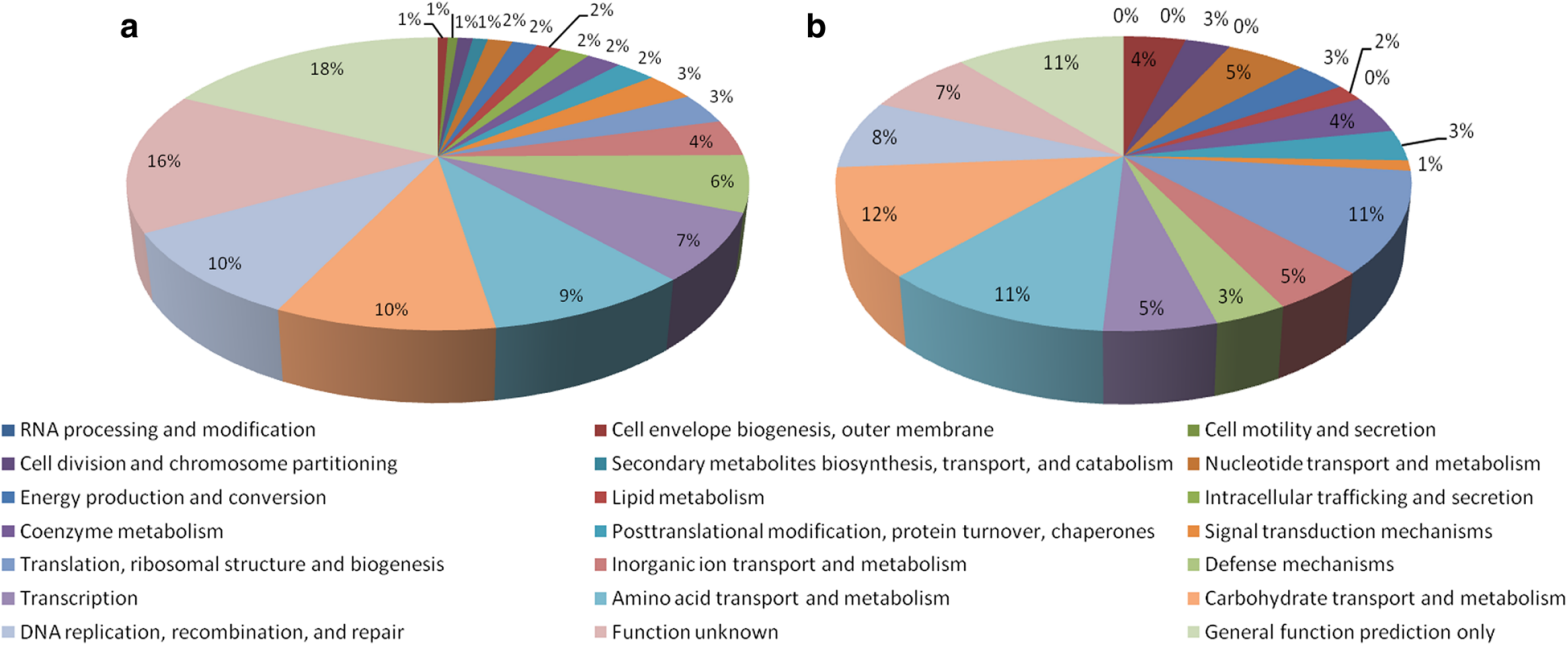
Upregulated genes after pH challenge in JDM301 and JDM301AR. These genes were grouped according to functional category. **a** A total of 322 genes were upregulated in JDM301 and **b** 263 genes in JDM301AR when cells were exposed to acid stress for 1.5 h

When JDM301 was grown at pH 3.5, a total of 322 genes were upregulated compared with that at normal medium (pH = 6.5) (Fig. 4a). When JDM301AR was cultured at pH 3.5, a total of 263 genes were upregulated, which were smaller in number than JDM301 (Fig. 4b). These genes were grouped according to functional category and the percent of each functional category was shown in Fig. 4. The genes encoding ammonium transporter (BLJ_0220) in JDM301 (Log_2_FC = 1.7) and JDM301AR (Log_2_FC = 1.1) were both upregulated under acid stress, which suggested that the import of ammonium is enhanced to neutralize proton. Additionally, cystathionine gamma-synthase (BLJ_1400) was also upregulated in JDM301 (Log_2_FC = 1.7) and JDM301AR (Log_2_FC = 2.0) (Additional file 2), which suggested that the ability to produce NH_3_ is promoted in both strains under acid stress.

### Different responses in peptidoglycan biosynthesis induced under pH challenge

A total of 11 genes (BLJ_0153, BLJ_0225, BLJ_0390, BLJ_1055, BLJ_1847, BLJ_2036, BLJ_1295, BLJ_1297, BLJ_1298, BLJ_1299, BLJ_1301) including a gene cluster (BLJ_1295-BLJ_1301) involved in peptidoglycan biosynthesis were upregulated at low pH in JDM301AR compared with normal condition (Additional file 2). However, only two of them (BLJ_0153, Log_2_FC = 1.2 and BLJ_1299, Log_2_FC = 1.2) were up-regulated by low pH in JDM301 (Additional file 2). The cell envelope and membrane (CEM) of bifidobacteria are mainly composed of peptidoglycan, lipid and exopolysaccharides. The CEM are probably the first targets of physicochemical stress [28]. These results suggested that the synthesis of peptidoglycan is further induced under acid stress in JDM301AR. Given that the genes of JDM301AR involved in peptidoglycan synthesis had been upregulated after successive batch cultures compared with JDM301 in normal condition, it can be inferred that the enhancement of peptidoglycan synthesis participates in acid stress adaptation of JDM301AR.

### Impact of low pH on the amino acid metabolism of JDM301 and JDM301AR

The deamination of branched-chain amino acid (BCAA) has been postulated as a mechanism to maintain bacterial internal pH [6]. Two genes (BLJ_1392, Log_2_FC = 1.5; BLJ_1393, Log_2_FC = 1.0) in an operon involved in the transport of branched-chain amino acids (BCAA) were upregulated by low pH in JDM301AR (Additional file 2). An apparent operon (BLJ_0094- BLJ_0097) containing genes involved in BCAA transport, showed significant upregulation after acid stress in JDM301 (Additional file 2). These results suggest that the two operons might be used to promote deamination of BCAA and help cells cope with acid stress in the two strains, respectively.

Previous report showed that there were glutamate-, arginine- and lysine-dependent acid resistance pathways in bacteria [29]. In our study, a total of 53 genes participating in amino acid metabolism were upregulated, in which 4 genes for glutamate metabolism, 3 genes for arginine biosynthesis and 11 genes for the biosynthesis of essential amino acid lysine were discovered in JDM301AR exposed in acid stress. No downregulated genes in amino acid metabolism were detected in JDM301AR after acid challenge. The l-lysine biosynthesis via DAP pathway (BLJ_0147, BLJ_0149, BLJ_0489, BLJ_1832, BLJ_1564, BLJ_1831, BLJ_1383 and BLJ_1843) was activated in JDM301AR (Additional file 2). During the process, the homologs of diaminopimelate decarboxylase (BLJ_1843, Log_2_FC = 1.6) were shown to specifically catalyze the decarboxylation of meso-diaminopimelate (meso-DAP) to l-lysine with a proton consumed from the cytoplasm [30]. For l-lysine biosynthesis via DAP pathway in parental strain, only 7 genes (BLJ_0147, BLJ_0149, BLJ_0489, BLJ_0490, BLJ_1299, BLJ_1383 and BLJ_1831) were upregulated after exposure to acid stress and the expression of BLJ_1843 was not changed (Additional file 2). The number of upregulated genes associated with amino acid metabolism in JDM301 was smaller (16 genes) than that of JDM301AR (Additional file 2). Furthermore, 8 genes involved in amino acid metabolism were downregulated in JDM301 (Additional file 2), when it was exposed to acid environment. Remarkably, among the downregulated genes, a gene cluster (including BLJ_0636-BLJ_0638, BLJ_0640 and BLJ_0642) encoding proteins responsible for arginine biosynthesis was found (Additional file 2). These results indicate that the organism’s ability to consume cellular proton may be improved by enhancing lysine and arginine biosynthesis in JDM301AR.

### Influence of low-pH on membrane fatty acid (FA)

**Fig. 5:**
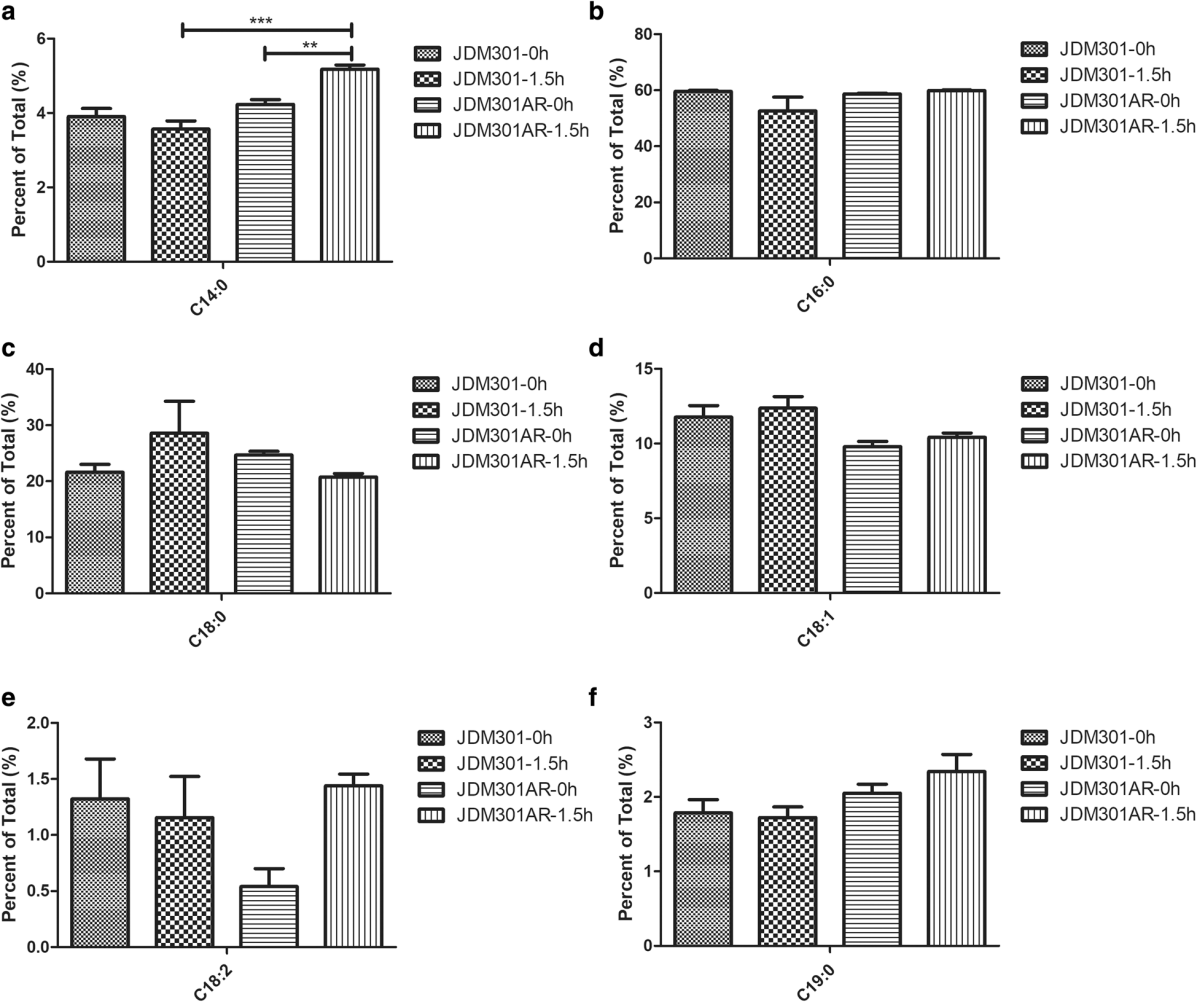
Membrane fatty acid composition for JDM301 and JDM301AR. The graphs showed data from cells grown in MRS without acid stress and cells exposed to acidified MRS (pH = 3.5) for 1.5 h. The percentages of FAs in the membrane of JDM301 and JDM301AR under normal condition (0 h) or acid stress (1.5 h) were shown. **a** The percentage of C14:0; **b** the percentage of C16:0; **c** the percentage of C18:0; **d** the percentage of C18:1; **e** the percentage of C18:2; **f** the percentage of C19:0. Error bars represent the standard error of the mean (SEM). **P < 0.01, ***P < 0.001

The modification of FA in cell membrane plays an important role in acid tolerance response. Therefore, the membrane FA composition of JDM301 or JDM301AR was investigated after acid exposure. The FA profiles of JDM301 or JDM301AR were all dominated by two major FA (C16:0 and C18:0) both in normal condition and acid stress. Analysis showed no significant change in membrane composition in JDM301 cells grown in acidic medium for 1.5 h compared with cells grown in control medium (Fig. 5). The strain JDM301AR showed a significant increase (p < 0.05) in C14:0 after exposure to acid stress (Fig. 5a). Furthermore, direct comparison between the two strains showed dramatic difference in cells exposed to acid stress. JDM301AR in acidic medium for 1.5 h had more C14:0 than JDM301 exposed to acid stress (Fig. 5a). Previous reports showed that bacterial capability of changing its membrane FA composition could help it adapt to pH stress [31, 32]. These results suggested that the increase of C14:0 may be helpful for JDM301AR to survive under acid stress.

In order to identify the basis for the differences in membrane lipid composition, the expression profiles of genes involved in FA metabolism were investigated in JDM301AR and JDM301 after exposure to acid stress. A total of 4 genes associated with FA metabolism were upregulated and no downregulated genes were found in response to acid stress in JDM301AR (Additional file 2). For JDM301, only 2 upregulated genes (BLJ_1756, Log_2_FC = 2.0 and BLJ_1809, Log_2_FC = 1.5) were detected and the two genes were also upregulated in JDM301AR (BLJ_1756, Log_2_FC = 1.4 and BLJ_1809, Log_2_FC = 1.0) under acid stress (Additional file 2). For JDM301AR, another two genes (BLJ_0565, Log_2_FC = 1.1 and BLJ_1105, Log_2_FC = 1.2) for FA metabolism were upregulated specifically in response to acid stress (Additional file 2). The reaction catalyzed by aldehyde dehydrogenase (ALDH) not only deactivates alkanals to convert them into FA, but also produces NADPH required for both the synthesis of FA and cell survival under stress conditions [33, 34]. The homolog of *ALDH* gene (BLJ_0565) was specifically upregulated in JDM301AR under acid stress and may be used by JDM301AR to promote incorporation of FA into the cytomembrane by deactivating alkanals to FA. In addition, NADPH can reduce oxidized glutathione (GSSG) to glutathione (GSH) [35]. The expression of 4 genes (BLJ_0511, Log_2_FC = 1.9; BLJ_0624, Log_2_FC = 2.8; BLJ_0916, Log_2_FC = 1.2 and BLJ_1324, Log_2_FC = 2.3) involved in glutathione metabolism was upregulated in this strain, while only one gene (BLJ_0511, Log_2_FC = 1.7) was up-regulated in JDM301. GSH could protect bacteria from acid stress, oxidative stress and osmotic stress by maintaining intracellular redox equilibrium [36, 37]. These results implied that BLJ_0565 may enhance cell survival ability under acid stress by promoting the production of FA and NADPH. The other specifically upregulated gene in JDM301AR, BLJ_1105 (Log_2_FC = 1.2) was predicted to encode a long-chain fatty acid coenzyme A (CoA) ligase. The gene was shown to activate exogenous long-chain fatty acids to incorporate into the cellular membrane, which might result in a different membrane lipid profile and promote JDM301AR survival under acid stress. Previous report showed that the mutation of long-chain fatty acid coenzyme A (CoA) ligase limited the ability of bifidobacterial strain to incorporate exogenous FA into its cytoplasmic membrane [38]. It was speculated that the up-regulated BLJ_0565 and BLJ_1105 may play important roles in the modification of membrane FA composition of JDM301AR after acid exposure.

## Conclusions

The transcriptional and physiological data in this study revealed that successive batch cultures under standard condition can induce the acquisition of acid resistance. Furthermore, the adaptive response provided this bacterium with cross-protection against osmotic and oxdative stress. Compared with its parental strain, some differences in how the resistant strain overcome the acidic environment were presented, which could reflect the adaptation of the resistant strain to acid stress. Moreover, a shift in the FA composition of cell membrane was found, when the acid resistant strain, JDM301AR was exposed to low-pH environment. These results may be associated with the specifically upregulated homologs of aldehyde dehydrogenase and long-chain fatty acyl-CoA ligase. Overall, as a response to low pH, the resistant strain may strengthening its resistance mainly by reinforcing amino acid metabolism, enhancing the generation and transport of NH_3_, modification of cell wall and cell member by remolding peptidoglycan biosynthesis and fatty acids metabolism. This study revealed the basis for understanding bifidobacterial acid tolerance response improved by successive batch cultures in normal condition without additional pressure.

## Methods

### Bacterial strains and growth conditions

*Bifidobacterium longum* JDM301, an industrial strain in China was used in this study. Bacteria were cultured in modified MRS broth (Difco, Leeuwarden, The Netherlands) supplemented with 0.05% (w/v) l-cysteine-HCl at 37 °C under anaerobic conditions (Don Whitley Scientific Limited, West Yorkshire, The England) in an atmosphere of 5% CO_2_–5% H_2_–90% N_2_.

### Isolation of acid-resistant derivative of JDM301 (JDM301AR)

Cells were inoculated in modified MRS at an initial pH of 6.5 and cultured under anaerobic condition for 24 h. After growth under anaerobic condition for 24 h, the cultures were adjusted to an optical density of 0.5 at 600 nm (OD_600_) and sub-cultured (10%, 0.3 ml in a total volume of 3 ml) in fresh modified MRS (pH = 6.5). These cultures were incubated at 37 °C for 24 h at the same condition for 150 repeats. Then the steps were repeated and colonies were isolated from the 150th repeats (Adapted strain, JDM301AR).

### Stress tolerance test

After 150 repeats, stress tolerance test was performed to reveal the potential difference of survival ability upon acid stress, oxidative or osmotic stress between JDM301AR and its parental strain JDM301. The cultures of *B. longum* JDM301 (Non-adapted strain, JDM301) and the isolated colonies of the 150th repeats (JDM301AR) were prepared by two successive transfers into modified MRS [10% inoculum (v/v)]. Cells cultured for about 16 h were used to perform stress tolerance tests. The survival ability was determined under acid stress (pH 3.5), oxidative stress (1.25 mM H_2_O_2_) or osmotic stress (0.5 M NaCl). The cultures were transferred into acidified liquid MRS medium (pH = 3.5) and inoculated under anaerobic condition at 37 °C for 1.5 h to test the survival ability under acid stress. Similarly, cultures were transferred into the MRS supplemented with 0.5 M NaCl or the MRS with the addition of 1.25 mM H_2_O_2_ to test the survival ability under oxidative stress and osmotic stress, respectively. After anaerobic incubation at 37 °C for 1.5 h, the surviving cells were counted by plate count on MRS agar medium.

### RNA isolation

Cultures from 3 ml samples incubated under the condition above were harvested by centrifugation. Cell pellets (~ 1 × 10^9^ cells) were suspended in 1 ml Trizol Reagent and disrupted with benchtop homogenizer (MP Biomedicals, California, USA). Then total RNA was extracted and the quantity of RNA was determined by a NanoDrop spectrophotometer (ThermoFisher Scientific, Waltham, MA). The quality of RNA was assayed using an Agilent 2100 bioanalyzer (Agilent Technologies, Waldbronn, Germany).

### RNA sequencing

The total RNA with RNA integrity number > 8.0 was treated with DNaseI (Takara, Janpan). Then mRNA was purified using a Dynabeads mRNA purification Kit (Life Technologies, USA). The cDNA library was constructed using NEBNext UltraTM RNA Library Pre Kit for Illumina (NEB, USA) according to manufacturer’ protocol. A cDNA library was sequenced using an Illumina HiseqTM2500 sequencer according to the manufacturer’ instructions. For each sample, about five million pair-end reads (2 × 150 bp) were obtained for further analysis. All sequenced reads were aligned to the genome of *B. longum* JDM301 and the sequences were deposited in NCBI Sequence Read Archive (SRA) database under accession number PRJNA555155. All these uniquely mapped reads were used to calculate the RPKM values (Reads per kilobase of exon per million mapped sequenced reads) of genes. It indicated the normalized gene expression level. MARS (MA-plot-based method with Random sampling model) in an R package DEGseq was used to identify differentially expressed genes (DEGs) from different samples, |FC| > 2; fdr < 0001 [39].

### RT-PCR assays

Validation of RNA sequencing data was performed by RT-PCR. The primers of selected genes were designed using Primer 5 based on the complete genome sequences of JDM301. The primers used were shown in additional file 3. Total RNA (2ug) was used to yield cDNA as template for RT-PCR using the Revert Aid First Strand cDNA Synthesis Kit (Fermentas, Waltham, USA). RT-PCR was performed using the Fast Start Universal SYBR Green Master (Rox, Roche) and using the following conditions: 95 °C for 10 min, followed by 40 cycles at 95 °C for 15 s, 58 °C for 30 s and 72 °C for 30 s. The 16S rDNA gene was used as an internal standard. Calculations were performed using the 2^−ΔΔCT^ method to determine the relative gene expression [40].

### Analysis of the fatty acid composition

The membrane fatty acid composition of the cells was determined by a previously reported method with a few modifications. Briefly, cells were grown in batch cultures under the condition above. Cells in 20 ml samples were collected by centrifugation and washed by phosphate-buffered saline. Then membrane fatty acids were isolated from the pelleted cells and identified using gas chromatography as previously reported [41, 42].

## Supplementary information

**Additional file 1.** List of genes involved in NH_3_ production, peptidoglycan synthesis, pantothenate and CoA biosynthesis and cell division and chromosome partitioning expressed differently in JDM301AR compared with JDM301 in normal condition.

**Additional file 2.** Differently expressed genes involved in peptidoglycan biosynthesis, metabolism and transport of amino acid and fatty acid metabolism in JDM301AR and JDM301 or in the two strains after exposure to acid stress.

**Additional file 3.** Primers used in RT-PCR for validation of RNA sequencing data.
