# Supplementary material for: Adaptational changes in physiological and transcriptional responses of Bifidobacterium longum involved in acid stress resistance after successive batch cultures
Source: Microb Cell Fact. 2019 Sep 12;18:156. doi: 10.1186/s12934-019-1206-x (PMC6743126; doi:10.1186/s12934-019-1206-x)
Supplement: Supplementary file 1 — Additional file 1. List of genes involved in NH3 production, peptidoglycan synthesis, pantothenate and CoA biosynthesis and cell division and chromosome partitioning expressed differently in JDM301AR compared with JDM301 in normal condition. [file 12934_2019_1206_MOESM1_ESM.docx]

| **Additional file 1** List of genes involved in NH_3_ production, peptidoglycan synthesis, pantothenate and CoA biosynthesis and cell division and chromosome partitioning expressed differently in JDM301AR compared with JDM301 in normal condition | | | |
| --- | --- | --- | --- |
| **Gene ID** | **Category** | **Predicted function** | **Log_2_FC^*^** |
| BLJ_1400 | NH3 production | MetC, cystathionine gamma-synthase | 1.6 |
| BLJ_1778 |  | Cystathionine beta-lyase | -10.1 |
| BLJ_1779 |  | Cystathionine gamma lyase | -9.0 |
| BLJ_1300 | Peptidoglycan synthesis | MurE, UDP-N-acetylmuramyl tripeptide synthase | 1.1 |
| BLJ_1301 |  | Peptidoglycan glycosyltransferase | 1.4 |
| BLJ_1302 |  | CheA, Chemotaxis protein histidine kinase and related kinases | 1.7 |
| BLJ_1303 |  | S-adenosylmethionine-dependent methyltransferase involved in cell envelope biogenesis | 1.2 |
| BLJ_0525 | Pantothenate and CoA biosynthesis | CoaX, type III pantothenate kinase | 1.4 |
| BLJ_0526 |  | CoaBC, phosphopantothenoylcysteine decarboxylase | 1.1 |
| BLJ_0527 |  | Extracellular solute-binding protein | 1.7 |
| BLJ_0528 |  | Binding-protein-dependent transport systems inner membrane component | 1.0 |
| BLJ_0529 |  | Binding-protein-dependent transport systems inner membrane component | 1.3 |
| BLJ_0530 |  | Peptide ABC transporter ATP-binding protein | 1.3 |
| BLJ_0531 |  | Peptide ABC transporter ATP-binding protein | 1.4 |
| BLJ_0092 | Cell division and chromosome partitioning | CrcB, Integral membrane protein possibly involved in chromosome condensation | -1.6 |
| BLJ_0093 |  | CrcB, Integral membrane protein possibly involved in chromosome condensation | -4.1 |
| BLJ_0135 |  | ATPases involved in chromosome partitioning | -3.1 |
| BLJ_0570 |  | ArsA, Predicted ATPase involved in chromosome partitioning | 1.1 |
| BLJ_0917 |  | FtsZ, Cell division GTPase | -2.4 |
| BLJ_1422 |  | MIH1, Mitotic inducer, protein phosphatase | -3.1 |
| BLJ_1879 |  | SulA, SOS-response cell division inhibitor, blocks FtsZ ring formation | 1.9 |

***FC, Fold change.**
