# Supplementary material for: Adaptational changes in physiological and transcriptional responses of Bifidobacterium longum involved in acid stress resistance after successive batch cultures
Source: Microb Cell Fact. 2019 Sep 12;18:156. doi: 10.1186/s12934-019-1206-x (PMC6743126; doi:10.1186/s12934-019-1206-x)
Supplement: Supplementary file 2 — Additional file 2. Differently expressed genes involved in peptidoglycan biosynthesis, metabolism and transport of amino acid and fatty acid metabolism in JDM301AR and JDM301 or in the two strains after exposure to acid stress. [file 12934_2019_1206_MOESM2_ESM.docx]

| **Additional file 2** Differently expressed genes involved in peptidoglycan biosynthesis, metabolism and transport of amino acid and fatty acid metabolism in JDM301AR and JDM301 or in the two strains after exposure to acid stress. | | | | |
| --- | --- | --- | --- | --- |
| **Gene ID** | **Category** | **Predicted function** | **JDM301AR (Log_2_FC^*^)** | **JDM301 (Log_2_FC^*^)** |
| BLJ_0153 | Peptidoglycan biosynthesis | UDP-N-acetylglucosamine 1-carboxyvinyltransferase | 2.3 | 1.2 |
| BLJ_0225 |  | UDP-N-acetylmuramoylalanine--D-glutamate ligase | 1.2 | ns^#^ |
| BLJ_0390 |  | UDP-N-acetylmuramoyl-L-alanyl-D-glutamate--2,6-diaminopimelate ligase | 1.0 | ns^#^ |
| BLJ_1055 |  | UDP-N-acetylmuramoyl-tripeptide--D-alanyl-D-alanine ligase | 2.3 | ns^#^ |
| BLJ_1295 |  | Undecaprenyl-diphosphatase | 1.3 | ns^#^ |
| BLJ_1297 |  | Phospho-N-acetylmuramoyl-pentapeptide-transferase | 1.9 | ns^#^ |
| BLJ_1298 |  | UDP-N-acetylglucosamine--N-acetylmuramyl-(pentapeptide) pyrophosphoryl-undecaprenol N-acetylglucosamine transferase | 2.0 | ns^#^ |
| BLJ_1299 |  | Putative peptidoglycan lipid II flippase | 1.6 | 1.1 |
| BLJ_1301 |  | Penicillin-binding protein 1A | 1.6 | ns^#^ |
| BLJ_1847 |  | Cell division protein FtsI | 1.1 | ns^#^ |
| BLJ_2036 |  | Peptidoglycan pentaglycine glycine transferase | 1.3 | ns^#^ |
| BLJ_0090 | Alanine, aspartate and glutamate metabolism | ADSS; adenylosuccinate synthase | ns^#^ | 1.3 |
| BLJ_0511 |  | GFPT; glucosamine--fructose-6-phosphate aminotransferase (isomerizing) | 1.9 | 1.7 |
| BLJ_0624 |  | GLUL; glutamine synthetase | 2.8 | ns^#^ |
| BLJ_0916 |  | Glutamate synthase (NADPH/NADH) large chain | 1.2 | ns^#^ |
| BLJ_1324 |  | GLUL; glutamine synthetase | 2.3 | ns^#^ |
| BLJ_0147 | Glycine, serine and threonine metabolism | Aspartate kinase | 1.1 | 2.0 |
| **Table 2** continued. | | | | |
| **Gene ID** | **Category** | **Predicted function** | **JDM301AR (Log_2_FC^*^)** | **JDM301 (Log_2_FC^*^)** |
| BLJ_0149 |  | Aspartate-semialdehyde dehydrogenase | 1.1 | 1.1 |
| BLJ_0443 |  | GCSH; glycine cleavage system H protein | 1.5 | ns^#^ |
| BLJ_0532 |  | Cystathionine beta-synthase | ns^#^ | 1.9 |
| BLJ_0590 |  | Threonine aldolase | 2.2 | 2.5 |
| BLJ_0626 |  | Dihydrolipoamide dehydrogenase | 1.4 | ns^#^ |
| BLJ_0670 |  | Threonine synthase | 1.0 | ns^#^ |
| BLJ_1306 |  | PHGDH; D-3-phosphoglycerate dehydrogenase | 1.3 | 1.3 |
| BLJ_1500 |  | PSPH; phosphoserine phosphatase | 1.2 | ns^#^ |
| BLJ_1668 |  | 2,3-bisphosphoglycerate-dependent phosphoglycerate mutase | 1.2 | ns^#^ |
| BLJ_1764 |  | PHGDH; D-3-phosphoglycerate dehydrogenase | 1.2 | ns^#^ |
| BLJ_0147 | Cysteine and methionine metabolism | Aspartate kinase | 1.1 | 2.0 |
| BLJ_0149 |  | Aspartate-semialdehyde dehydrogenase | 1.1 | 1.1 |
| BLJ_0268 |  | Homoserine O-succinyltransferase | 2.3 | 1.7 |
| BLJ_0532 |  | Cystathionine beta-synthase | ns^#^ | 1.9 |
| BLJ_0536 |  | S-ribosylhomocysteine lyase | ns^#^ | -1.5 |
| BLJ_0799 |  | O-acetylhomoserine (thiol)-lyase | 3.4 | 2.5 |
| BLJ_0946 |  | 5-methyltetrahydropteroyltriglutamate--homocysteine methyltransferase | 1.1 | ns^#^ |
| BLJ_1400 |  | MetC, cystathionine gamma-synthase | 2.0 | 1.7 |
| BLJ_1846 |  | Cystathione beta-lyase | ns^#^ | 1.6 |
| BLJ_0446 | Valine, leucine and isoleucine degradation and biosynthesis | 3-isopropylmalate dehydrogenase | 1.4 | ns^#^ |
| BLJ_0565 |  | Aldehyde dehydrogenase (NAD+) | 1.1 | ns^#^ |
| BLJ_0626 |  | Dihydrolipoamide dehydrogenase | 1.4 | ns^#^ |
| **Table 2** continued. | | | | |
| **Gene ID** | **Category** | **Predicted function** | **JDM301AR (Log_2_FC^*^)** | **JDM301 (Log_2_FC^*^)** |
| BLJ_0147 | Lysine biosynthesis | Aspartate kinase | 1.1 | 2.0 |
| BLJ_0149 |  | Aspartate-semialdehyde dehydrogenase | 1.1 | 1.1 |
| BLJ_0225 |  | UDP-N-acetylmuramoyl-L-alanyl-D-glutamate--2,6-diaminopimelate ligase | 1.2 | ns^#^ |
| BLJ_0489 |  | 4-hydroxy-tetrahydrodipicolinate reductase | 1.1 | 1.7 |
| BLJ_0490 |  | 4-hydroxy-tetrahydrodipicolinate synthase | ns^#^ | 1.0 |
| BLJ_0544 |  | 2-aminoadipate transaminase | 1.3 | ns^#^ |
| BLJ_1299 |  | UDP-N-acetylmuramoyl-tripeptide--D-alanyl-D-alanine ligase | 1.6 | 1.2 |
| BLJ_1564 |  | 2,3,4,5-tetrahydropyridine-2-carboxylate N-succinyltransferase | 1.3 | ns^#^ |
| BLJ_1831 |  | Succinyl-diaminopimelate desuccinylase | 1.7 | 1.6 |
| BLJ_1383 |  | Diaminopimelate epimerase | 1.0 | 1.0 |
| BLJ_1832 |  | 4-hydroxy-tetrahydrodipicolinate synthase | 1.3 | ns^#^ |
| BLJ_1843 |  | Diaminopimelate decarboxylase | 1.6 | ns^#^ |
| BLJ_0565 | Lysine degradation | Aldehyde dehydrogenase (NAD+) | 1.1 | ns^#^ |
| BLJ_0624 | Arginine biosynthesis | GLUL; glutamine synthetase | 2.8 | ns^#^ |
| BLJ_0636 |  | N-acetyl-gamma-glutamyl-phosphate reductase | ns^#^ | -2.1 |
| BLJ_0637 |  | Glutamate N-acetyltransferase/amino-acid N-acetyltransferase | ns^#^ | -1.1 |
| BLJ_0638 |  | Acetylglutamate kinase | ns^#^ | -1.4 |
| BLJ_0640 |  | Ornithine carbamoyltransferase | ns^#^ | -1.1 |
| BLJ_0642 |  | ASS1; argininosuccinate synthase | ns^#^ | -1.7 |
| BLJ_1324 |  | GLUL; glutamine synthetase | 2.3 | ns^#^ |
| BLJ_0565 | Arginine and proline metabolism | Aldehyde dehydrogenase (NAD+) | 1.1 | ns^#^ |
| BLJ_0251 |  | Diamine N-acetyltransferase | 1.1 | ns^#^ |
| BLJ_0805 |  | Pyrroline-5-carboxylate reductase | 2.0 | ns^#^ |
| BLJ_0980 |  | N-carbamoylputrescine amidase | ns^#^ | 1.3 |
| BLJ_0565 | Histidine metabolism | Aldehyde dehydrogenase (NAD+) | 1.1 | ns^#^ |
| BLJ_1095 |  | Phosphoribosyl-AMP cyclohydrolase | 1.0 | 1.6 |
| **Table 2** continued. | | | | |
| **Gene ID** | **Category** | **Predicted function** | **JDM301AR (Log_2_FC^*^)** | **JDM301 (Log_2_FC^*^)** |
| BLJ_1325 |  | Phosphoribosylformimino-5-aminoimidazole carboxamide ribotide isomerase | ns^#^ | -1.1 |
| BLJ_1756 | Tyrosine metabolism | Acetaldehyde dehydrogenase/alcohol dehydrogenase | 1.4 | 2.0 |
| BLJ_1415 | Phenylalanine metabolism | 3-hydroxybutyryl-CoA dehydrogenase | 1.2 | 1.4 |
| BLJ_0565 | Tryptophan metabolism | Aldehyde dehydrogenase (NAD+) | 1.1 | ns^#^ |
| BLJ_0754 | Phenylalanine, tyrosine and tryptophan biosynthesis | 3-phosphoshikimate 1-carboxyvinyltransferase | 1.1 | ns^#^ |
| BLJ_0565 | Metabolism of other amino acids | Aldehyde dehydrogenase (NAD+) | 1.1 | ns^#^ |
| BLJ_0210 |  | 6-phosphogluconate dehydrogenase | 1.7 | 1.0 |
| BLJ_0213 |  | Glucose-6-phosphate 1-dehydrogenase | 1.2 | ns^#^ |
| BLJ_0492 |  | Aminopeptidase N | 1.4 | ns^#^ |
| BLJ_0946 |  | 5-methyltetrahydropteroyltriglutamate--homocysteine methyltransferase | 1.1 | ns^#^ |
| BLJ_1107 |  | Isocitrate dehydrogenase | 2.3 | ns^#^ |
| BLJ_1297 |  | UDP-N-acetylmuramoylalanine--D-glutamate ligase | 1.9 | ns^#^ |
| BLJ_1400 |  | MetC,cystathionine gamma-synthase | 2.0 | 1.7 |
| BLJ_1944 |  | Methionyl-tRNA synthetase | 1.2 | ns^#^ |
| BLJ_1846 |  | Cystathione beta-lyase | ns^#^ | 1.6 |
| BLJ_2037 |  | Thioredoxin reductase (NADPH) | ns^#^ | 1.1 |
| BLJ_0094 | Amino acid transport system | Branched-chain amino acid transport system permease protein | ns^#^ | 1.5 |
| BLJ_0095 |  | Branched-chain amino acid transport system permease protein | ns^#^ | 1.6 |
| BLJ_0096 |  | Branched-chain amino acid transport system ATP-binding protein | ns^#^ | 1.9 |
| **Table 2** continued. | | | | |
| **Gene ID** | **Category** | **Predicted function** | **JDM301AR (Log_2_FC^*^)** | **JDM301 (Log_2_FC^*^)** |
|  |  |  |  |  |
|  |  |  |  |  |
| BLJ_0097 |  | Branched-chain amino acid transport system ATP-binding protein | ns^#^ | 1.5 |
| BLJ_0098 |  | Neutral amino acid transport system substrate-binding protein | 1.6 | 2.3 |
| BLJ_0775 |  | D-methionine transport system permease protein | 1.3 | ns^#^ |
| BLJ_1387 |  | Cystine transport system permease protein | ns^#^ | 1.0 |
| BLJ_1388 |  | Cystine transport system substrate-binding protein | ns^#^ | 1.4 |
| BLJ_1389 |  | Putative amino-acid transport system ATP-binding protein | ns^#^ | 1.7 |
| BLJ_1392 |  | Branched-chain amino acid transport system ATP-binding protein | 1.5 | ns^#^ |
| BLJ_1393 |  | Branched-chain amino acid transport system permease protein | 1.0 | ns^#^ |
| BLJ_1394 |  | Branched-chain amino acid transport system substrate-binding protein | ns^#^ | 2.0 |
| BLJ_0565 | Fatty acid metabolism | Aldehyde dehydrogenase (NAD+) | 1.1 | ns^#^ |
| BLJ_1105 |  | Long-chain acyl-CoA synthetase | 1.7 | ns^#^ |
| BLJ_1756 |  | Acetaldehyde dehydrogenase/alcohol dehydrogenase | 1.4 | 2.0 |
| BLJ_1809 |  | Acetyl-CoA carboxylase, biotin carboxylase subunit | 1.0 | 1.5 |
| #, ns, not significantly regulated after acid exposure. | | | | |

*, FC, Fold change.
