# Supplementary material for: Adaptational changes in physiological and transcriptional responses of Bifidobacterium longum involved in acid stress resistance after successive batch cultures
Source: Microb Cell Fact. 2019 Sep 12;18:156. doi: 10.1186/s12934-019-1206-x (PMC6743126; doi:10.1186/s12934-019-1206-x)
Supplement: Supplementary file 3 — Additional file 3. Primers used in RT-PCR for validation of RNA sequencing data. [file 12934_2019_1206_MOESM3_ESM.docx]

| **Additional file 3. Primers used in RT-PCR for validation of RNA sequencing data** | | | | |
| --- | --- | --- | --- | --- |
| **Gene ID** | **Direction** | **Primer sequence** | **Length**  **(bp)** | **Source or reference** |
| BLJ_0295 | Sense | GCACATTGCAACGCGACCCC | 159 | This work |
|  | Antisense | ATGGCCAGCACGGCCACATC |  |  |
| BLJ_0343 | Sense | GGGGAGCGCGATGGCAAAGT | 209 | This work |
|  | Antisense | CTCCCCCGTACGCGACCAGA |  |  |
| BLJ_0536 | Sense | TCACACCAAGGTCAAGGCAC | 130 | This work |
|  | Antisense | ATGCGATGAACTCCAACGAT |  |  |
| BLJ_0620 | Sense | CCTGACGCTGGTGGACGAT | 183 | This work |
|  | Antisense | GGGCAGCACGGTGACATT |  |  |
| BLJ_0999 | Sense | GTCAGCAAGGACGAGGAAAG | 176 | This work |
|  | Antisense | CAGGTTCAATCGCAGGGTC |  |  |
| BLJ_1575 | Sense | AGATTCAGGCTACGGACAT | 242 | This work |
|  | Antisense | CGGCAGTTGGAAGAAGTC |  |  |
| BLJ_1640 | Sense | CGTAAGTTCGTGGCTGTC | 287 | This work |
|  | Antisense | GCTAGGTTCTTGGCAATCAT |  |  |
| BLJ_1693 | Sense | CGAGGGGCTGAACAACCAT | 196 | This work |
|  | Antisense | CGGAGAACACAAGCGGAAG |  |  |
| BLJ_1977 | Sense | AAGGAAAAGGGCGTTGAGGT | 164 | This work |
|  | Antisense | CGTATTGGACATATCGGCGGT |  |  |
